# The Flipped Classroom: A Critical Appraisal

**DOI:** 10.5811/westjem.2019.2.40979

**Published:** 2019-04-16

**Authors:** Aaron S. Kraut, Rodney Omron, Holly Caretta-Weyer, Jaime Jordan, David Manthey, Stephen J. Wolf, Lainie M. Yarris, Stephen Johnson, Josh Kornegay

**Affiliations:** * University of Wisconsin School of Medicine and Public Health, Berbee Walsh Department of Emergency Medicine, Madison, Wisconsin; † Johns Hopkins School of Medicine, Department of Emergency Medicine, Baltimore, Maryland; ‡ Stanford University School of Medicine, Department of Emergency Medicine, Palo Alto, California; § David Geffen School of Medicine at UCLA, Department of Emergency Medicine, Los Angeles, California; ¶ Wake Forest School of Medicine, Department of Emergency Medicine, Winston-Salem, North Carolina; || Denver Health Medical Center, Department of Emergency Medicine, Denver, Colorado; # Oregon Health and & Science University, Department of Emergency Medicine, Portland, Oregon; ** University of Wisconsin School of Medicine and Public Health, Library Services, Madison, Wisconsin; †† Oregon Health and & Science University, Department of Emergency Medicine, Portland, Oregon

## Abstract

**Introduction:**

The objective of this study was to review and critically appraise the medical education literature pertaining to a flipped-classroom (FC) education model, and to highlight influential papers that inform our current understanding of the role of the FC in medical education.

**Methods:**

A search of the English-language literature querying Education Resources Information Center (ERIC), PsychINFO, PubMed, and Scopus identified 296 papers related to the FC using either quantitative, qualitative, or review methods. Two reviewers independently screened each category of publications using previously established exclusion criteria. Eight reviewers then independently scored the remaining 54 publications using either a qualitative, quantitative, or review-paper scoring system. Each scoring system consisted of nine criteria and used parallel metrics that have been previously used in critical appraisals of education research.

**Results:**

A total of 54 papers (33 quantitative, four qualitative, and 17 review) on FC met a priori criteria for inclusion and were critically appraised and reviewed. The top 10 highest scoring articles (five quantitative studies, two qualitative studies, and three review papers) are summarized in this article.

**Conclusion:**

This installment of the Council of Emergency Medicine Residency Directors (CORD) Academy Critical Appraisal series highlights 10 papers that describe the current state of literature on the flipped classroom, including an analysis of the benefits and drawbacks of an FC approach, practical implications for emergency medicine educators, and next steps for future research.

## INTRODUCTION

The Council of Emergency Medicine Residency Directors (CORD) Academy Critical Appraisal Series approaches important, relevant educational problems with a rigorous literature review, systematic article scoring, and summary of the top papers to provide an understanding of the topic and describe practical implications for emergency medicine (EM) educators. In this installment of the series, we address the flipped classroom (FC) model in medical education. Traditional classroom (TC) didactics and lectures remain a common format in medical education despite well described limitations.^1^,^2^ The FC approach has been suggested as one technique to overcome some of these limitations.

The working definition of the FC describes a technique where foundational knowledge is acquired independently by a learner prior to a classroom encounter. This knowledge is then applied during in-person interactions facilitated by an instructor, often in the form of case-based discussions, allowing for higher-level problem solving.^3^,^4^ Components of FC may also be applied in a more heterogeneous “blended learning” approach, where online or asynchronous activities may supplement foundational concepts that are taught in a traditional face-to-face learning environment.^5^ For the purposes of this critical appraisal, we will define FC to include instructional techniques that incorporate independent knowledge acquisition prior to a classroom encounter focused on the application of that knowledge. Despite the recent interest in the FC, little is known about optimal implementation strategies and the impact of this model on learning outcomes.^6^

This review applies a previously published method to search and critically appraise the literature regarding the FC model in medical education.^7^,^8^ The objective of this appraisal was to summarize and highlight the top scoring papers in medical education regarding the FC, as well as present implications for EM educators and suggest future areas for research surrounding this important topic.

## METHODS

### Article Identification

A research librarian performed the literature search, querying Education Resources Information Center (ERIC), PsychINFO, PubMed, and Scopus to identify articles – limited to the English language – mapped to the medical subject headings terms “flipped classroom,” or “inverted classroom,” or “flipped learning,” or “blended learning.” Although the primary aim was to review FC papers, we found these terms are often used interchangeably in the literature, and instructional techniques universally fall somewhere on the spectrum between true “flipped classroom” and “blended learning.”^5^ Therefore, all terms were included for the initial search. An initial search found 296 papers using either quantitative (hypothesis-testing or observational investigations of educational interventions), qualitative (exploring important phenomena in EM education), or review methods. The literature search was conducted in March 2017.

**Population Health Research Capsule**

What do we already know about this issue?

*The flipped classroom (FC) approach to didactics is becoming increasingly popular among medical educators and has several ideal applications*.

What was the research question?

*To critically appraise the literature to help define the role of the FC in medical education*.

What was the major finding of the study?

*Key themes from the top 10 papers on the FC are summarized*.

How does this improve population health?

*This study provides guidance to medical educators looking to adopt an FC approach in the education of the next generation of physicians*.

### Inclusion and Exclusion Criteria

We included publications relevant to the education of medical students, residents, attending physicians, and other healthcare professionals. Medical education studies were defined as hypothesis-testing investigations, evaluations of educational interventions, explorations of educational problems using either quantitative or qualitative methods, or review papers that synthesized existing literature to provide a new understanding of the topic. We excluded publications if 1) they were not considered to be peer-reviewed research (such as opinion pieces, commentaries, or curricula descriptions without outcomes data); 2) upon further review, the topic of the paper was not FC, but rather small-group interactive learning in the classroom setting or other teaching strategies; 3) they were not relevant to EM learners (such as reports on education of prehospital personnel or international studies that could not be generalized to EM training outside of the country in which they were performed); 4) they were single-site survey studies of individual curricula; 5) they were studies that examined outcomes limited to an expected learning effect without a comparison group; or 6) they were studies where the abstract or manuscript could not be obtained from the libraries of any of the authors.

### Data Collection

**Figure 1:**
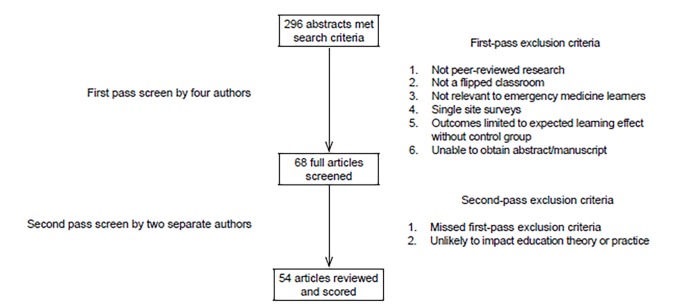
Selection process for articles that focus on the flipped classroom model in medical education.

Four authors (AK, JK, LY, and HCW) independently screened 296 abstracts from all retrieved publications and applied the exclusion criteria. After the first pass, they identified 68 manuscripts. Two separate authors (DM and RO) then performed a second-pass exclusion, reviewing all remaining manuscripts and excluding those that either had an exclusion criterion that was missed, or were not felt to have the potential to impact education theory or practice (e.g., studies that supported a widely accepted theory and lacked novelty, or those with methods that would be difficult to replicate for the majority of educators). All differences in opinion were resolved by direct discussion, which included the first author of this appraisal and negotiated consensus. We maintained retrieved publications in a Microsoft Excel 2010 (Microsoft Inc., Redmond, Washington) database. After complete review, 54 final publications were made electronically available to all reviewers (Figure 1).

### Scoring

The publications were first assigned to a scoring system based on whether they were primarily quantitative studies, qualitative studies, or review articles. The quantitative studies used scoring criteria that were developed in 2009 and then continually optimized and iteratively modified since then.^9^ Quantitative studies were scored in nine domains for a maximum total score of 25 points. The domains included the following: introduction (0–3 points); measurement (0–4 points); data collection (0–4 points); data analysis (0–3 points); discussion (0–3 points); limitations (0–2 points); innovation (0–2 points); generalizability (0–2 points); and clarity of writing (0–2 points). Each of the domains were scored based on predefined criteria to make scoring as objective as possible.

Using a previously published parallel scoring sheet developed based on guidelines for qualitative research and subsequently updated to reflect newer recommendations for increasing rigor and iteratively modified since then, we assessed and scored qualitative studies in nine domains, parallel to those applied to the quantitative studies, for a maximum total score of 25 points.^10^ These also included the domains of measurement, data collection, and data analysis criteria, as defined specifically for high-quality qualitative research. Review papers were scored according to criteria established through an iterative process for the inaugural critical appraisal work, which includes the same nine domains as the quantitative and qualitative scoring instruments.^8^

To establish response process validity, pairs of authors read each scoring instrument aloud to ensure agreement in the interpretation of each scoring category. To establish reliability, each author read one quantitative, one qualitative, and one review paper and scored them using the appropriate scoring instrument, with good agreement. Inter-rater reliability by Shrout-Fleiss interclass correlation for absolute agreement was 0.646 across multiple raters.

All scoring scales are presented in Supplemental Tables 1, 2, and 3.

### Data Analysis

**Figure 2:**
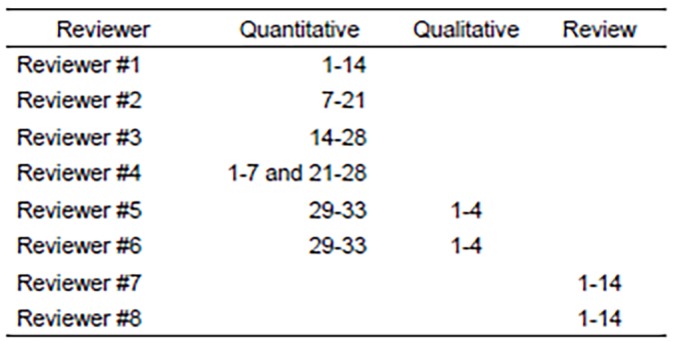
Article review breakdown by author.

Reviewers were excluded from scoring publications in which there was deemed to be significant conflict of interest (own publication, own institution, or had a vested interest in the authors or work). We separated publications by category (quantitative, qualitative, or review), and authors were assigned to small groups to read and score a comparable number of papers in a particular category. Assignments were based on methodological expertise of the scorer, and we ensured that all qualitative and review papers were scored by the same two reviewers, and that each paper was independently scored by two separate authors. Each reviewer first read a sample paper in their assigned category, scored it independently, and then read aloud the scoring instrument to other members of the group to ensure items were interpreted and scored consistently. Figure 2 illustrates the author breakdown for publication review. Each reviewer independently reviewed and scored each publication in his or her assigned category, except those excluded for conflict of interest.

A total rating score was calculated for each article and entered into a spreadsheet using Microsoft Excel 2010 (Microsoft Inc., Redmond, Washington). Average scores for each article were calculated and all categories were analyzed by the first and last author to determine a natural cutoff that separated the top articles from the rest. For all three categories, quantitative, qualitative and review, a score of 18/25 represented a cutoff below which the majority of the papers clustered. Therefore, a decision was made to include the five quantitative, two qualitative, and three review papers that scored above 18. Finally, we included several additional studies as supplemental resources for readers interested in learning more about FC development and implementation. The decision to include these additional papers was based upon consensus discussion between authors AK and JK.

### Theme synthesis

After the top scoring papers were identified, two authors (AK and JK) performed a constant comparative qualitative analysis of the themes represented through independently coding the themes and subthemes of each paper, and then conducting an iterative round of discussions to reach consensus on four prevailing and consistent themes and best practices.

## RESULTS

A total of 296 papers satisfied the search criteria, 54 of which met the inclusion criteria (33 quantitative studies, four qualitative studies, and 17 review papers). All 54 papers were critically appraised and scored independently by two reviewers. Five quantitative studies met criteria as methodologically superior publications with a potential to impact current educational practices, with a range of mean scores from 18 to 20.5 (maximum 25 points).^11^–^15^ Two qualitative studies met criteria as superior publications with a range of mean scores from 18 to 21 (maximum 25 points).^16^,^17^ Three review papers met criteria as superior publications with a range of mean scores from 19.5 to 23.5 (maximum 25 points).^18^–^20^ All top papers are listed in alphabetical order by study design and summarized in Tables 1–3. Finally, two additional studies – one quantitative 21 and one review^22^ – were noted to be useful references by reviewers and are included as supplements in Table 4. The identified consensus themes and best practices around implementation of an FC along with suggested future areas for research are summarized in Table 5.

## DISCUSSION

Although there has been a great deal of enthusiasm for the FC model, educators unfamiliar with this instructional approach may struggle to identify appropriate applications and potential drawbacks. Furthermore, potential adopters of the FC should be armed with a thorough understanding of its relative efficacy for knowledge dissemination when compared to a “traditional” lecture approach. In our literature review and critical appraisal, several themes emerge that help define the current state of the FC in medical education.

### A Flipped Classroom or Blended Learning Approach is Effective for Procedural Learning

One common application of the FC model across the continuum of medical education is in procedural education. Procedural instruction has traditionally occurred via traditional classroom modalities including lecture-based demonstrations or in-person skills stations. As blended learning and the FC model have become more prevalent, educators have successfully implemented these innovations in the delivery of procedural skills training ranging from Advanced Cardiac and Pediatric Advanced Life Support courses to more focused training sessions such as instructing students in suturing techniques and general surgical procedures.^11^,^17^

Lehman et al.^11^ identified that learners who received a procedural curriculum via a blended learning model demonstrated superior procedural knowledge, higher procedural quality via objective measures, and a higher adherence to the clinical care algorithms when compared to a control group who received instruction via traditional lecture and skills station teaching. Similarly, Liebert et al.^17^ reported that procedural skills-based video sessions were among the highest rated components of a new FC curriculum among students enrolled in a surgical clerkship.

Importantly, this reveals that FC and blended learning curricula that use meaningful and interactive pre-work (that prime the learner to think critically about the rationale for and mentally rehearse the steps of a procedure) may better prepare learners to perform procedural skills compared to traditional teaching methods.

We acknowledge that the FC approach may be equally effective for non-procedural learning, although this has been studied less frequently, perhaps because procedural learning is particularly well suited to an FC model.

### Students in a Flipped Classroom Setting May Learn More Than Students in a Traditional Classroom Setting

Beyond the realm of procedural skills education, there is emerging evidence that an FC model may outperform traditional lecture-based education in a much broader context, both in terms of knowledge and skills acquisition. While this effect is not universally reported, the FC model appears to be at least non-inferior to the standardized lecture-based educational format.^11^,^14^ Learners particularly valued the FC model’s ability to promote active learning, engagement, and facilitation of peer and faculty interaction; thus, it is important that in-class activities be designed with this in mind.^16^,^20^ Activities such as simulation sessions, clinical cases, problem-based learning, team-based learning, and discussion activities align well with these values and were particularly appreciated.^16^,^17^,^20^

### The Flipped Classroom Model is Beneficial for Learning Higher Cognition Tasks

When faculty are aiming to teach high-order Bloom’s objectives, such as analysis or evaluation, TC or isolated e-learning alone may not be the most effective approach. With these more advanced objectives, an FC or blended learning approach using both TC and e-learning seems to be preferred and result in greater learning. This is particularly true when accompanied by in-class active learning, such as case-based or self-assessment exercises.^12^,^15^,^19^ This large positive effect favoring the FC or blended learning model is seen when comparing those two modalities to TC or e-learning alone and proves to be consistent across disciplines and course settings.^18^

Furthermore, relatively recent work by Morton et al.^12^ suggests that FC is better suited to teach analysis or application of knowledge than memorization of general facts. When the FC model is used in a manner that builds upon foundational concepts or previously-mastered facts, it may facilitate focused learning in these higher-order skills by optimizing a learner’s germane cognitive load. However, when used to teach basic concepts that are easily grasped, the FC model may serve only to increase the extraneous cognitive load placed on the student and not increase their mastery of the subject.^12^

### Learners Are More Engaged with Flipped Classroom, but Satisfaction Depends Largely on Teacher Prep Work

When applied in the appropriate context, the FC model seems to promote superior student engagement as compared to the TC model. According to O’Connor et al., “subjects who participated in the flipped learning cohorts had greater interest in learning, increased enjoyment, and higher task value than the traditional didactic instruction cohorts.”^13^ The finding that FC increases learner engagement is consistent with educational theory that posits that learners who take an active role in their learning may be more motivated to learn, and instruction that builds upon a common foundation may be more engaging.^23^

Like any curriculum, use of the FC model also requires high-quality, pre-class material and in-class learning activities, aligned with course goals and objectives and matched to learner level and needs, to be successful. While learners generally viewed the FC model positively, they also noted that design and implementation of the curriculum was important for outcomes. Learners valued pre-class materials that were specifically designed for the FC model, were easy to access and use, and included content that was concise, relevant, well organized, and delivered by a variety of modalities.^16^,^17^,^20^ High-quality videos of approximately 20–30 minutes duration were particularly valued as a means of delivering this content.^16^ While learners generally appreciated the self-directed aspect of the FC model, they also called attention to the importance of realistic expectations in terms of workload of pre-class material to avoid cognitive overload or lack of completion of assignments in preparation for the in-class component.^16^

It is also important that instructors be well trained in the FC model and consistent in their delivery and expectations, as deviation from this approach can negatively impact learners.^16^ Careful attention must be paid to provide an adequate transition between pre-class and in-class work while avoiding both redundancy and introduction of completely new material in the in-class portion in order for the sessions to be most effective.^16^

As with any other program of study, instructors must also ensure that assessment tools are in line with the goals and objectives of the course and curricular model. All of this requires deliberate and purposeful planning and delivery on the part of course directors and instructors wishing to implement the FC model. In fact, our review found that preparation in terms of cost and faculty time may be significant and this should be considered prior to implementing the FC model. It may be beneficial to secure sources of funding, support staff, and infrastructure such as high-speed internet capacity and information technology support in advance to assist in successful implementation.^22^

### Next Steps for Research

With a deeper understanding of both the advantages and limitations of the FC model, education scholars are poised to begin exploring the next steps and identify future research questions to understand how best to employ this educational strategy. Further studies are needed to explore which platforms are most effective for presenting pre-course portions of the FC model. The determination of which procedural skills are best taught through FC needs further elucidation. Additionally, while preliminary research suggests that higher-level skills such as analysis and application are better suited to FC methods than acquisition of facts, further exploration of the specific learning outcomes that are optimally suited to FC curricula would be useful to educators. While this appraisal demonstrates that FC is associated with a higher level of learner engagement than TC, it will be important to determine if this level of engagement directly translates to a higher level of knowledge transfer and learner performance than other methods. Finally, additional studies comparing outcomes among FC, TC, or a blended approach incorporating both of these strategies are greatly needed to advance our understanding on the best practices for classroom didactics.

## LIMITATIONS

This critical appraisal had several important limitations. Although the scoring instruments have been previously published, threats to validity remain, as it is possible the instruments did not measure what we intended them to measure. In addition, we specifically highlighted methodologically rigorous papers with our scoring system. It is possible that papers that were not as methodologically rigorous may still have resulted in important theoretical findings and could have been missed by our method.

As we aimed to identify the papers that rose to the top, rather than selecting a score cutoff in advance, we planned on evaluating the scores to identify a natural cutoff. We selected a cutoff of 18/25 as the majority of paper scores clustered below this, allowing us to highlight and analyze in more depth a small number of superior papers. While this cutoff was consistent with that used for prior similar critical appraisals, papers just below the cutoff may also have had important results that were missed.

Furthermore, several of the exclusion criteria used in this appraisal are admittedly subjective. By limiting our reviewed manuscripts to those that were deemed relevant to emergency medicine learners and that were felt to have the potential to impact education theory and practice, we may have excluded important studies. Additionally, while we elected to exclude all single-site survey studies of individual courses that might be limited in their generalizability, there was not a similar exclusion of single-site qualitative or mixed-methods studies, which may be prone to similar biases.

Finally, we note that this appraisal includes a disproportionally large number of studies on an FC application to procedural teaching as opposed to knowledge or non-procedural skills acquisition. This likely reflects a publication bias toward FC procedural curricula in the medical literature, as these are inherently easier to implement and study than curricula targeted at knowledge or non-procedural skills acquisition.

## CONCLUSION

Our understanding of the role of the FC in medical education has steadily grown over the last 10–15 years since it was first introduced. This CORD Academy critical appraisal highlights several rigorous and relevant publications on FC theory and application, in order to serve as both a resource and summary for educators.

## Supplementary Information







## Figures and Tables

**Table 1:** Top-scoring quantitative papers.

Citation	Aims	Findings	Contributions to current knowledge
Bonnes SL et al.^14^ Flipping the Quality Improvement Classroom in Residency Education	To develop and validate an instrument to measure resident perceptions of a quality improvement (QI) curriculum delivered via an FC vs a TC approach	QI knowledge increased significantly in those residents exposed to the FC vs the TC curriculum. Residents who had no experience with an FC environment had larger improvement of scores than those who had previous FC experience, suggesting novelty as a factor.	Pre-class activity and in-class application serve to enhance learning. This reinforces the concept of cognitive load and the requirement for the instructor to be present for the application, not the acquisition, of new knowledge.
Lehmann R et al.^11^ Improving Pediatric Basic Life Support Performance Through Blended Learning With Web-Based Virtual Patients: Randomized Controlled Trial	To investigate the impact of a blended learning approach, including web-based virtual patients (VPs) and standard pediatric basic life support (PBLS) training, on procedural knowledge, objective performance in a simulated case, and trainee self-assessment.	Procedural knowledge in the blended learning group was significantly better than that of the control group after the preparation period. After the hands-on training, the blended learning group showed significantly better adherence to a resuscitation algorithm and better procedural quality of PBLS in objective measures than did the control group.	For complex procedures, a blended learning approach may be superior to traditional teaching methods. VPs may be helpful in bridging the gap between knowledge and practice.
Morton DA et al.^12^ Measuring the Impact of the Flipped Anatomy Classroom: The importance of Categorizing an Assessment by Bloom’s Taxonomy	To determine whether FC instruction is superior to TC instruction for learning gross anatomy.	The FC method significantly improved students’ ability to analyze material on a final examination relative to the TC. No difference was observed in FC and TC students’ ability to recall or recognize (knowledge level) material on a final examination.	Students in an FC setting may perform better than those in a TC on assessments requiring higher cognition (e.g., analysis), but the same on those requiring lower cognition (e.g., memorization and recall)
O’Connor EE et al.^13^ Flipping Radiology Education Right Side Up	To compare the effects of FC vs TC on students’ academic achievement, task value, and achievement emotions.	Assessment of task value and achievement emotions showed greater task value, increased enjoyment, and decreased boredom with FC as compared to TC.	The positive emotional effects of FC on medical students’ motivational beliefs and achievement emotions can enhance academic performance. The FC approach provides medical students with the opportunity to develop self-directed learning skills while also providing opportunities to solidify already acquired knowledge and concepts through active learning strategies.
Rui Z et al.^15^ Friend or Foe? Flipped Classroom for Undergraduate Electrocardiogram Learning: A Randomized Controlled Study	To observe whether FC teaching improved learner performance as compared to a TC model. To investigate the attitudes of learners and teachers toward the FC.	The students in the FC group scored significantly higher than those in the TC group. The majority of students held positive attitudes toward the FC, but also supported the TC method. Teachers invested more time and energy into the FC, but also felt it to have greater learning effects than the TC.	While an FC model appeared more effective for student learning, it required significantly more teacher time and effort for material design than a TC model.

*FC*, flipped classroom; *TC*, traditional classroom.

**Table 2:** Top scoring qualitative papers.

Citation	Aims	Findings	Contribution to current knowledge
Khanova J et al.^16^ Student Experiences Across Multiple Flipped Courses in a Single Curriculum	To examine student perspectives of the FC model across multiple courses.	Students liked the FC model and identified multiple benefits, but these were conditional on effective implementation. They noted challenges of an increased workload and the importance of high-quality instructional materials, alignment of pre-class and in-class learning activities, and the critical role of the instructor.	This study provides insight into the learner experience of the FC model across multiple courses and highlights multiple elements that may be important for effective design and implementation of this model.
Liebert CA et al.^17^ Student Perceptions of a Simulation-based Flipped Classroom for the Surgery Clerkship: A Mixed-Methods Study	To evaluate learner perceptions of a simulation-based FC curriculum in a third- year surgical clerkship.	Learners viewed the curriculum very positively and valued succinct videos, use of multiple teaching modalities, and content that was high yield and relevant. Students felt that this model created an interactive and engaging environment that promoted self-directed learning and accountability. Perceived benefits of the curriculum included preparation for clinical rotations and knowledge tests, improved comfort with clinical skills, and positive interactions with peers and faculty.	This study demonstrates that an FC model can be incorporated into a third-year surgical clerkship, and that it is well received by learners. The authors recommend best practices for implementation of an FC into a core clerkship based on study results and their personal experience.

*FC*, flipped classroom; *TC*, traditional classroom.

**Table 3:** Top scoring review papers.

Citation	Aims	Findings	Contribution to current knowledge
Liu Q et al.^18^ The Effectiveness of Blended Learning in Health Professions: Systematic Review and Meta-Analysis	To assess the effectiveness of blended learning for health professionals compared with a TC model or purely e-learning model.	A blended learning approach was often more effective than non-blended instruction (either traditional lecture or purely e-learning) with regard to learner knowledge acquisition. Unfortunately, the significant heterogeneity of studies included in the meta-analysis limits generalizability.	This systematic review and meta-analysis supports the concept that a blended learning model is at least as efficacious as either a TC or purely e-learning model with regard to learner knowledge acquisition.
McCutcheon K et al.^19^ A Systematic Review Evaluating the Impact of Online or Blended Learning vs. Face-to-Face Learning of Clinical Skills in Undergraduate Nurse Education	To determine if the use of an online or blended learning paradigm has potential to enhance the teaching of clinical skills in undergraduate nursing education.	Online or blended learning methods were as effective as TC methods when teaching clinical skill to nursing students.	This review highlights the important role that online and blended learning approaches have for teaching technical clinical skills when compared to face-to-face modalities. Online or blended instructional approaches may allow for more learner and instructor flexibility when neither party is tied to a traditional classroom setting.
Ramnanan CJ et al.^20^ Advances in Medical Education and Practice: Student Perceptions of the Flipped Classroom	To identify trends in learner perception of the pre-class and in-class phases of the FC approach and to identify the impact of the FC method on learning.	The most commonly applied methods for pre-class and in-class activities in an FC model are video-based learning and case-based learning. The FC methodology appears well received by learners as it has been demonstrated to increase motivation, engagement and attendance. Although learners perceive that the FC model leads to improvements in their knowledge base relateive to the TC model, evidence for this is mixed.	This review highlights important trends in the development of FC learning models as they pertain to early learners. It further demonstrates the high satisfaction rates of this method with learners, although it is still unclear whether an FC approach leads to improvements in knowledge acquisiton when compared to a TC model.

*FC*, flipped classroom; *TC*, traditional classroom.

**Table 4:** Additional resource papers.

Citation	Aims	Findings	Contribution to current knowledge
Heitz C et al.^21^ Does the Concept of the “Flipped Classroom” Extend to the Emergency Medicine Clinical Clerkship?	To determine whether clerkship students achieve better mastery of educational objectives when an FC approach to clerkship is used as opposed to a TC model.	There was no observed difference in level of mastery of clerkship educational objectives using the FC approach (asynchronous modules before clinical shifts) vs the TC approach to clerkship learning.	There are many barriers to using an FC model to prepare emergency medicine clerkship students for “themed clinical shifts” including difficulty in students adhering to the set protocol. Additionally, it is does not appear that the FC model helps students to achieve a higher level of mastery than the TC model.
O’Flaherty J et al.^22^ The Use of Flipped Classrooms in Higher Education: A Scoping Review	To provide a review of relevant research on the FC including how key aspects contribute to its effectiveness as a learning modality.	Core features of the FC approach include content in advance (generally recorded lectures) educator awareness of level of student understanding, higher level learning in classroom setting significant time investment for faculty to create asynchronous learning resources trend toward improved test scores and improved opportunities for students to develop teamwork and communication skills in FC model vs TC model, although paucity of high-quality data and absence of demonstrated educational benefit in long term apparent lack of pedagogical understanding of how to operationalize FC from traditional teaching model.	This resources serves as an excellent review of concepts integral to the success of the FC model and includes suggestions for additional measures of student engagement, a hallmark of success in the FC model.

*FC*, flipped classroom; *TC*, traditional classroom.

**Table 5:** Consensus themes and best practices.

Themes and associated references	Current understanding	Areas of future research
FC and Procedural Learning^11^,^17^	FC and blended learning models may result in greater procedural competency and knowledge as well as greater satisfaction on the part of learners when compared with TC model of instruction.	What is the best pre-course approach for flipped classroom procedural teaching? Which procedures lend themselves best to a FC approach?
FC Better for Learning than TC^11^,^12^,^14^	FC is at least non-inferior to TC in terms of general knowledge acquisition on the part of learners, and may be superior for teaching analysis and application of concepts.	Is FC superior to TC or simply non-inferior? What aspects of the FC approach seem to help most when teaching higher-level concepts? Do learners simply spend more time with the material, or is in-person application of knowledge with faculty guidance the key?
FC Excels with Higher Cognition Tasks^12^,^15^,^18^,^19^	FC helps to optimize the germane cognitive load of the learner to outperform TC for tasks requiring analysis of information, such as case-based learning.	Which approach has the best outcomes when comparing among blended learning, FC, and TC?
Learners More Engaged with FC, but Satisfaction Depends Largely on Teacher Prep Work^13^,^16^,^17^,^20^,^23^	FC promotes higher task value and greater interest in learning than TC.	Does learner engagement directly translate to improved knowledge transfer?
	FC preparation materials must be concise, well organized, easy to access, and designed specifically for the FC.	What are the best ways to objectively evaluate learner engagement and perceptions of different learning modalities?

*FC*, flipped classroom; *TC*, traditional classroom.
