# Supplementary material for: The Flipped Classroom: A Critical Appraisal
Source: West J Emerg Med. 2019 Apr 16;20(3):527–36. doi: 10.5811/westjem.2019.2.40979 (PMC6526887; doi:10.5811/westjem.2019.2.40979)
Supplement: Supplementary file 1 [file wjem-20-527-s001.doc]

**Supplemental Table 1.** Quantitative scoring sheet.

**EM Educational Research Scoring Sheet: Quantitative Research**

| **Domain** | **Item** | | | **Item**  **score** | **Maximum**  **domain score** |  |
| --- | --- | --- | --- | --- | --- | --- |
| Introduction | | | |  | 3 |  |
|  | | 1. Give one point for each criterion met | |  |  |  |
|  |  |  | Appropriate description of background literature | 1 |  |  |
|  |  |  | Clearly frame the problem with defined outcome measures | 1 |  |  |
|  |  |  | Clear objective / hypothesis | 1 |  |  |
| Measurement (add methodology + groups) | | | |  | 4 |  |
|  | | 1. Methodology: Give a point for each criterion met | |  |  |  |
|  |  |  | No pre-test, post-test | 0 |  |  |
|  |  |  | Has a post-test | 1 |  |  |
|  |  |  | Has a pre-test and post-test | 1 |  |  |
|  |  | 2. Groups: Give a point for each criterion met | | |  |  |
|  |  |  | Both experimental and control group | 1 |  |  |
|  |  |  | Random assignment to groups | 1 |  |  |
| Data collection (add institutions + response rate) | | | |  | 4 |  |
|  | | 1. Institutions - Give a point for each criterion met | |  |  |  |
|  |  |  | Single institution | 0 |  |  |
|  |  |  | At least 2 institutions | 1 |  |  |
|  |  |  | More than 2 institutions | 1 |  |  |
|  |  |  | * a survey of many institutions should get both points |  |  |  |
|  |  | 2. Response Rate - Give a point for each criterion met | |  |  |  |
|  |  |  | Response rate <50% or not reported | 0 |  |  |
|  |  |  | Response rate ≥50% | 1 |  |  |
|  |  |  | Response rate ≥75% | 1 |  |  |
| Data analysis (add appropriateness + sophistication) | | | |  | 3 |  |
|  | | 1. Appropriateness - Give a point for each criterion met | |  |  |  |
|  |  |  | Data analysis inappropriate for study design or type of data | 0 |  |  |
|  |  |  | Data analysis appropriate for study design and type of data | 1 |  |  |
|  |  | 2. Sophistication - Give a point for each criterion met | |  |  |  |
|  |  |  | Descriptive analysis only | 0 |  |  |
|  |  |  | Beyond descriptive analysis | 1 |  |  |
|  | |  | Includes power analysis | 1 |  |  |

| Discussion | | |  | 3 |  |
| --- | --- | --- | --- | --- | --- |
|  | 1. Give one point for each criterion met | |  |  |  |
|  |  | Data supports conclusion | 1 |  |  |
|  |  | Conclusion clearly addresses hypothesis / objective | 1 |  |  |
|  |  | Conclusions placed in context of literature | 1 |  |  |
| Limitations | | |  | 2 |  |
|  | 1. Assign a score | |  |  |  |
|  |  | Limitations not identified accurately | 0 |  |  |
|  |  | Some limitations identified | 1 |  |  |
|  |  | Limitations well addressed | 2 |  |  |
| Innovation of project | | |  | 2 |  |
|  | 1. Assign a score | |  |  |  |
|  |  | Subjects “liked” intervention | 0 |  |  |
|  |  | Subjects learned something from intervention | 1 |  |  |
|  |  | Subjects changed behavior after intervention | 2 |  |  |
| Generalizability of project | | |  | 2 |  |
|  | 1. Assign a score | |  |  |  |
|  |  | Would be difficult to replicate at my program | 0 |  |  |
|  |  | Could implement at my program with additional effort/resources | 1 |  |  |
|  |  | Could be easily implemented at my program tomorrow | 2 |  |  |
| Clarity of writing | | |  | 2 |  |
|  | 1. Assign a score | |  |  |  |
|  |  | Verbose- at times difficulty to follow | 0 |  |  |
|  |  | Average for scientific manuscript | 1 |  |  |
|  |  | Clear, concise writing | 2 |  |  |
| **Total** |  |  |  | 25 |  |
