# Supplementary material for: The Flipped Classroom: A Critical Appraisal
Source: West J Emerg Med. 2019 Apr 16;20(3):527–36. doi: 10.5811/westjem.2019.2.40979 (PMC6526887; doi:10.5811/westjem.2019.2.40979)
Supplement: Supplementary file 2 [file wjem-20-527-s002.doc]

**Supplemental Table 2.** Qualitative scoring sheet.

**EM Educational Research Scoring Sheet: Qualitative Research**

| **Domain** | **Item** | | | **Item**  **Score** | **Maximum**  **Domain Score** |  |
| --- | --- | --- | --- | --- | --- | --- |
| Introduction | | | |  | 3 |  |
|  | | 1. Give one point for each criterion met | |  |  |  |
|  |  |  | Appropriate description of background literature | 1 |  |  |
|  |  |  | Clearly frame the problem with defined outcome measures | 1 |  |  |
|  |  |  | Clear study purpose | 1 |  |  |
| Measurement (add methodology + sampling) | | | |  | 3 |  |
|  | | 1. Methodology: Give a point for each criterion met | |  |  |  |
|  |  |  | Clear description of qualitative approach (e.g. ethnography, grounded theory, phenomenology, etc) | 1 |  |  |
|  |  |  | Method (observation, interviews, focus groups, etc) appropriate for study purpose | 1 |  |  |
|  |  | 2. Sampling of study participants: | |  |  |  |
|  |  |  | Sampling strategy well described and appropriate | 1 |  |  |
|  |  |  |  |  |  |  |
| Data collection | | | |  | 3 |  |
|  | | 1. Data collection methods – give a point for each criterion met | |  |  |  |
|  |  |  | Detailed description of data collection method | 1 |  |  |
|  |  |  | Description of instrument development | 1 |  |  |
|  |  |  | Description of instrument piloting | 1 |  |  |
|  |  |  |  |  |  |  |
|  |  |  |  |  |  |  |
| Data Analysis | | | |  | 5 |  |
|  | | 1. Sophistication of data analysis: Give a point for each criterion met | |  |  |  |
|  |  |  | Clear, reproducible “audit trail” documenting systematic procedure for analysis | 1 |  |  |
|  |  |  | Data saturation through a systematic iterative process of analysis | 1 |  |  |
|  |  |  | Addressed contradictory responses | 1 |  |  |
|  |  |  | Incorporated validation strategies (e.g., member checking, triangulation) | 1 |  |  |
|  |  |  | Addressed reflexivity (impact of researcher’s background, position, biases on study) | 1 |  |  |

| Discussion | | |  | 3 |  |
| --- | --- | --- | --- | --- | --- |
|  | 1. Give one point for each criterion met | |  |  |  |
|  |  | Clear summary of main findings | 1 |  |  |
|  |  | Conclusions placed in context of literature | 1 |  |  |
|  |  | Discussion of how findings should be interpreted/applied/or direct next steps (without overreaching) | 1 |  |  |
| Limitations | | |  | 2 |  |
|  | 1. Assign a score | |  |  |  |
|  |  | Limitations not identified accurately | 0 |  |  |
|  |  | Some limitations identified | 1 |  |  |
|  |  | Limitations well addressed | 2 |  |  |
| Novelty of project | | |  | 2 |  |
|  | 1. Assign a score | |  |  |  |
|  |  | Does not add to current knowledge | 0 |  |  |
|  |  | Adds somewhat to current knowledge | 1 |  |  |
|  |  | Significant contribution to what is known | 2 |  |  |
| Generalizability of project | | |  | 2 |  |
|  | 1. Assign a score | |  |  |  |
|  |  | Would be difficult to replicate at my program | 0 |  |  |
|  |  | Could implement at my program with additional effort/resources | 1 |  |  |
|  |  | Could be easily implemented at my program tomorrow | 2 |  |  |
| Clarity of Writing | | |  | 2 |  |
|  | 1. Assign a score | |  |  |  |
|  |  | Verbose- at times difficulty to follow | 0 |  |  |
|  |  | Average for scientific manuscript | 1 |  |  |
|  |  | Clear, concise writing | 2 |  |  |
| **Total** |  |  |  | 25 |  |
