# Supplementary material for: The Flipped Classroom: A Critical Appraisal
Source: West J Emerg Med. 2019 Apr 16;20(3):527–36. doi: 10.5811/westjem.2019.2.40979 (PMC6526887; doi:10.5811/westjem.2019.2.40979)
Supplement: Supplementary file 3 [file wjem-20-527-s003.docx]

**Supplemental Table 3.** Review scoring sheet.

**EM Educational Research Scoring Sheet: Review Papers**

| **Domain** | **Item** | | | **Item**  **score** | **Maximum**  **domain score** |  |
| --- | --- | --- | --- | --- | --- | --- |
| Introduction | | | |  | 3 |  |
|  | | 1. Give one point for each criterion met | |  |  |  |
|  |  |  | Appropriate description of background literature | 1 |  |  |
|  |  |  | Clearly frame the problem with defined outcome measures | 1 |  |  |
|  |  |  | Clear aim of the review | 1 |  |  |
| Measurement (add methodology + sampling) | | | |  | 3 |  |
|  | | 1. Methodology: Give a point for each criterion met | |  |  |  |
|  |  |  | Appropriate for study question | 1 |  |  |
|  |  | 2. Sampling of study participants: Give a point for each criterion met | |  |  |  |
|  |  |  | Appropriate inclusion/exclusion criteria for papers | 1 |  |  |
|  |  |  | Described thorough and appropriate literature search strategy | 1 |  |  |
| Data collection (review method rigor, assign a score) | | | |  | 3 |  |
|  | |  | Opinion piece | 0 |  |  |
|  |  |  | Thoughtful literature review, or expert consensus (using structured consensus-building approach) | 1 |  |  |
|  |  |  | Critical appraisal, using standardized scoring | 2 |  |  |
|  |  |  | Systematic review or metaanalysis | 3 |  |  |
| Data analysis | | | |  | 5 |  |
|  | | 1. Sophistication of data analysis: Give a point for each criterion met | |  |  |  |
|  |  |  | Uses a set protocol for paper selection | 1 |  |  |
|  |  |  | Each paper scored by at least 2 authors | 1 |  |  |
|  |  |  | Appropriate data abstraction description | 1 |  |  |
|  |  |  | Statistical analysis of agreement included | 1 |  |  |
|  |  |  | Description of how conflicts were resolved | 1 |  |  |

| Discussion | | |  | 3 |  |
| --- | --- | --- | --- | --- | --- |
|  | 1. Give one point for each criterion met | |  |  |  |
|  |  | Data supports conclusion | 1 |  |  |
|  |  | Conclusion clearly addresses objective | 1 |  |  |
|  |  | Conclusions placed in context of literature | 1 |  |  |
| Limitations | | |  | 2 |  |
|  | 1. Assign a score | |  |  |  |
|  |  | Limitations not identified accurately | 0 |  |  |
|  |  | Some limitations identified | 1 |  |  |
|  |  | Limitations well addressed | 2 |  |  |
| Novelty of review | | |  | 2 |  |
|  | 1. Assign a score | |  |  |  |
|  |  | Does not add to current knowledge | 0 |  |  |
|  |  | Adds as a synthesis of what is known, but no new knowledge | 1 |  |  |
|  |  | Synthesizes existing knowledge, and lends conclusions which move understanding forward | 2 |  |  |
| Generalizability of project | | |  | 2 |  |
|  | 1. Assign a score | |  |  |  |
|  |  | Would be difficult to replicate at my program | 0 |  |  |
|  |  | Could implement at my program with additional effort/resources | 1 |  |  |
|  |  | Could be easily implemented at my program tomorrow | 2 |  |  |
| Clarity of writing | | |  | 2 |  |
|  | 1. Assign a score | |  |  |  |
|  |  | Verbose- at times difficulty to follow | 0 |  |  |
|  |  | Average for scientific manuscript | 1 |  |  |
|  |  | Clear, concise writing | 2 |  |  |
| **Total** |  |  |  | 25 |  |
